# Supplementary material for: Type-2 innate signals are dispensable for skeletal muscle regeneration and pathology linked to Duchenne muscular dystrophy
Source: EMBO Rep. 2025 Feb 3;26(5):1406–21. doi: 10.1038/s44319-025-00383-y (PMC11894123; doi:10.1038/s44319-025-00383-y)
Supplement: Supplementary file 7 — Expanded View Figures [file 44319_2025_383_MOESM7_ESM.pdf]

Expanded View Figures

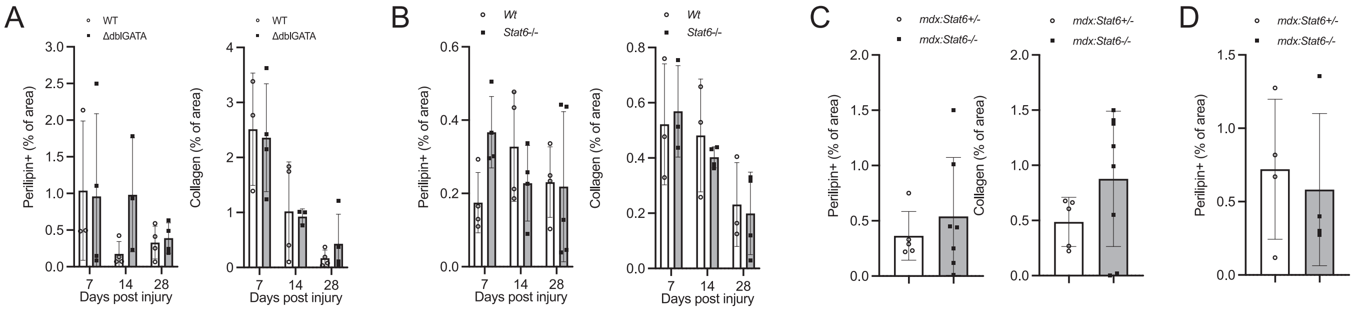

Figure EV1. Adipocyte and collagen deposition analysis.

Quantification of % perilipin and % collagen in TA muscle sections *Tibialis anterior* (TA) muscles of wild-type (*Wt*) or ΔdblGATA mice at 7-, 14-, and 28-days post injury after muscle injury with barium chloride ($BaCl_2$) (**A**). Quantification of % perilipin and % collagen in TA muscle sections muscles of wild-type (*Wt*) or *Stat6−/−* mice at 7-, 14-, and 28-days post injury after muscle injury with barium chloride ($BaCl_2$) (**B**). At age 3 months, the right TA of *mdx:Stat6+/−* and *mdx:Stat6−/−* mice were micro-damaged (MD).Tissues were collected at 14 weeks of age. Quantification of % perilipin and % collagen in MD TA muscle sections (**C**). Quantification of % perilipin in 10 months old *mdx:Stat6+/−* and *mdx:Stat6−/−* mice (**D**). Number of mice per group: $n = 3$–4. Error bars represent SD.

 © The Author(s)

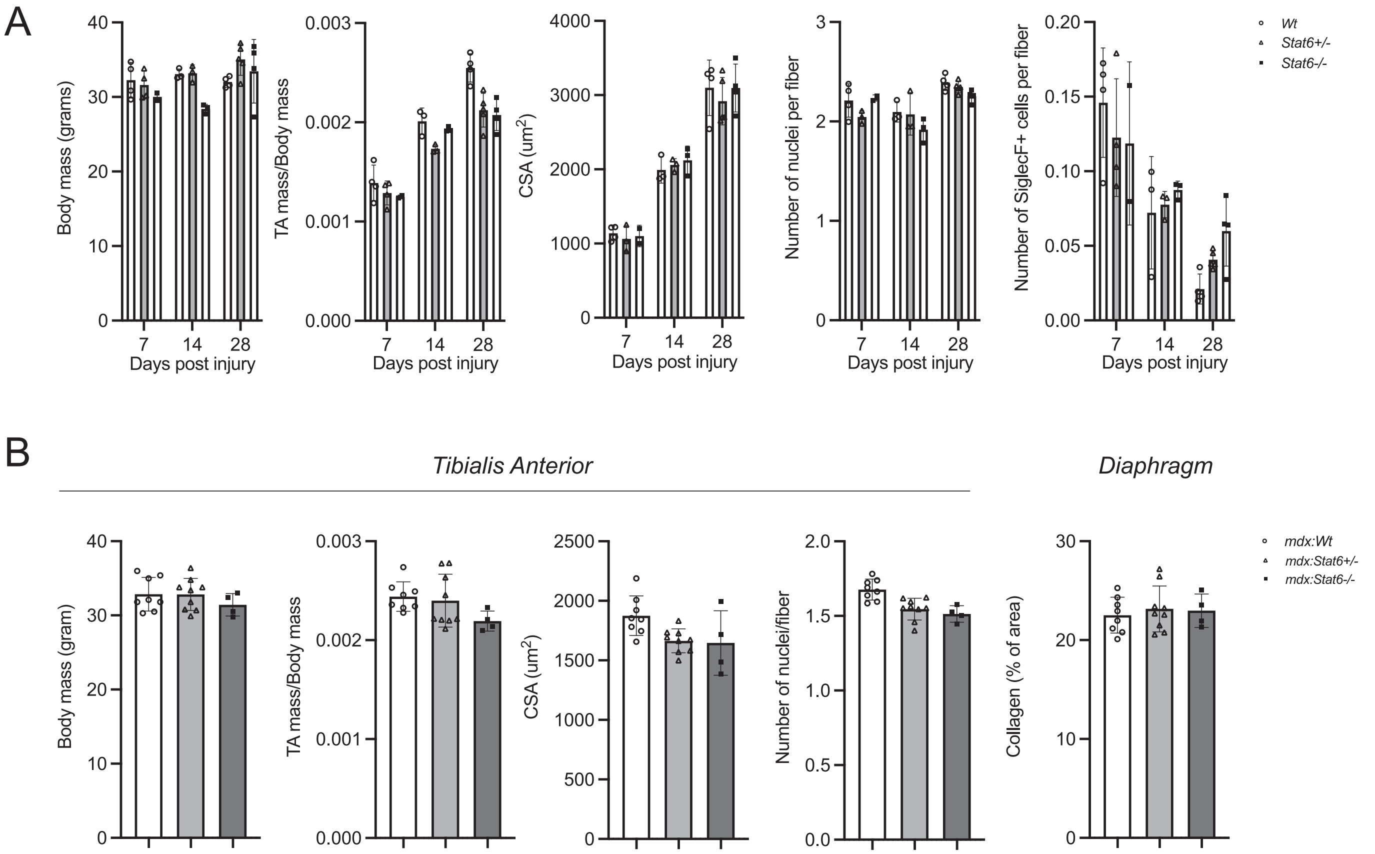

Figure EV2. Analysis of intermediate loss of STAT6.

Tibialis anterior (TA) muscles of wild-type (*Wt*), *Stat6+/−* or *Stat6−/−* mice were injured with barium chloride ($BaCl_2$) and body mass, TA mass (normalized to body mass), cross-sectional area (CSA), number of nuclei per fiber and number of SiglecF+ cells was quantified to assess muscle regeneration at 7-, 14-, and 28-days post injury (**A**). At age 3 months, mdx, *mdx:Stat6+/−* and *mdx:Stat6−/−* mice were compared in terms of body mass, TA/body mass, CSA and nuclei per fiber as well as % collagen deposition in the diaphragm (**B**). Number of mice per group: $n = 3–7$. Error bars represent SD.

© The Author(s)

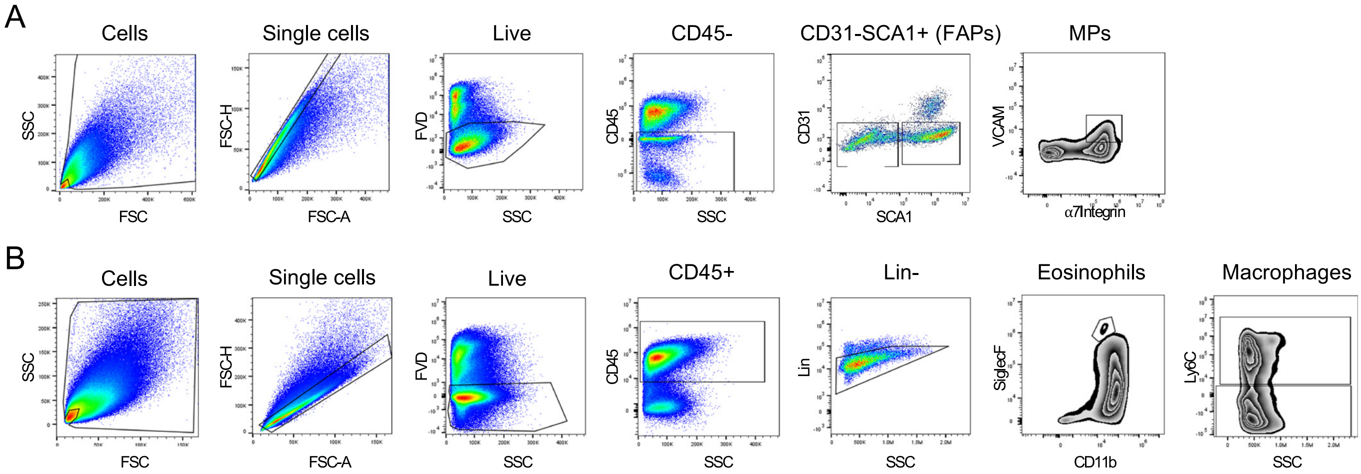

Figure EV3. Representative flow cytometry gating strategies.

Muscle resident cells gating strategy (**A**). Eosinophil and macrophage gating strategy (**B**). FVD fixable viability dye, Lin lineage.

 © The Author(s)
